# Association between workaholism, vital exhaustion, and hair cortisol concentrations among teachers: A longitudinal study testing the moderation effect of neuroticism

**DOI:** 10.3389/fpsyg.2022.1046573

**Published:** 2022-12-15

**Authors:** Alexander Wettstein, Sandra Schneider, Gabriel Jenni, Martin grosse Holtforth, Wolfgang Tschacher, Roberto La Marca

**Affiliations:** ^1^Department of Research and Development, University of Teacher Education Bern, Bern, Switzerland; ^2^Department of Psychology, Clinical Psychology and Psychotherapy, University of Bern, Bern, Switzerland; ^3^Department of Neurology, Psychosomatic Medicine, Bern University Hospital, Bern, Switzerland; ^4^Experimental Psychology Division, University Clinic for Psychiatry and Psychotherapy, University of Bern, Bern, Switzerland; ^5^Centre for Stress-Related Disorders, Clinica Holistica Engiadina, Susch, Switzerland; ^6^Department of Clinical Psychology and Psychotherapy, University of Zurich, Zürich, Switzerland

**Keywords:** workaholism, neuroticism, hair cortisol, vital exhaustion, teacher stress

## Abstract

**Introduction:**

Workaholism in teachers is characterized by the willingness to work until exhausted and may be associated with various adverse health outcomes as well as high economic costs. The present study examines the association between workaholism, vital exhaustion (VE), and hair cortisol concentration (HCC) as indicators of chronic stress. In addition, this study explores the moderating role of the personality trait neuroticism on the relationship between workaholism and chronic stress indicators, i.e., VE and HCC.

**Methods:**

Forty-two Swiss teachers (28 females; *M*_age_ = 39.66, SD = 11.99) completed questionnaires assessing VE (Maastricht Vital Exhaustion Questionnaire), workaholism (Measure of Coping Capacity Questionnaire), and neuroticism (Big-Five Inventory). Together with VE, HCC was assessed twice, with a one year lag.

**Results:**

Both workaholism and neuroticism were positively associated with VE at both time points but not with HCC. Moderation analyses revealed a positive relationship between workaholism and VE in teachers with high neuroticism, while no such association was observed in teachers with low neuroticism. No associations were found between self-reports and HCC.

**Discussion:**

These findings emphasize the importance of considering individual characteristics when investigating VE. Further research is necessary to investigate the applicability of HCC as a biomarker of chronic stress in the context of work.

## Introduction

In the teaching profession, the growing demands from policymakers and expectations from society, as well as the increasing number of tasks and bureaucratic work for which teachers lack time and resources, may lead to high external work pressure and chronic work overload, both during and after teachers’ workday (Apple, 1989; Kokkinos, 2007; Ballet and Kelchtermans, 2009; Isabel et al., 2021). When job demands exceed job resources, and certain individual predispositions are present, an obsessive need to work may occur, namely workaholism (Schaufeli, 2016; Tabak et al., 2021). Workaholism is characterized as a form of addiction that comprises the dimensions of affect (enjoying work but feeling guilty and anxious when not working), cognition (obsessing about work), and behavior (working excessive hours, even at home; Ng et al., 2007). The term workaholism was coined by Oates (1971), who described it as “the compulsion or the uncontrollable need to work incessantly” (p. 11), referring to the behavioral and cognitive components of alcoholism that may result in various stress-related health outcomes (Preckel et al., 2005; Clark et al., 2016).

Personality traits can influence how individuals perceive their work and how invested they become (Kokkinos, 2007; Schaufeli, 2016; Perera et al., 2018; Tabak et al., 2021). A growing body of research suggests that workaholism is positively related to neuroticism, as neuroticism may increase the risk of developing an obsessive need or dependence on work (Clark et al., 2016; Schaufeli, 2016). The Five-Factor Model (Costa and McCrae, 1992) describes neuroticism as being vulnerable to psychological stress, prone to unrealistic beliefs, and unable to control urges and successfully cope with stress. Furthermore, teachers with high neuroticism scores have been shown to be at higher risk for burnout based on situational anxiety related to challenging student behavior and classroom management, self-doubt, self-criticism, and work-related rumination (McIlveen and Perera, 2016).

Like neuroticism, workaholism was found to be positively related to burnout (Ng et al., 2007; Andreassen, 2014; Clark et al., 2016). A study by Nie and Sun (2016) found significant correlations between workaholism, job burnout, and depression in Chinese university teachers emphasizing how personal life and health can be affected when work becomes the priority in life. In addition, previous studies show an association between overcommitment to work (Preckel et al., 2005) and excessive workload (Schnorpfeil et al., 2002) with vital exhaustion (VE). The concept of VE has received increasing research attention for its adverse effects on health (Kop et al., 1996; Prescott et al., 2003; Balog and Konkolÿ, 2019). Characteristics of VE include excessive fatigue, lack of mental and physical energy, feelings of demoralization, and irritability (Appels and Mulder, 1989). Though the underlying causes of VE are still not fully known, it is thought that VE emerges when personal resources required to adapt to stress are depleted and can thus be considered a consequence of perceived chronic stress and burnout (Bellingrath et al., 2008; Schoch et al., 2018). Furthermore, VE has been shown to be a significant and independent risk factor for cardiovascular disease in men (Appels and Mulder, 1989). Kudielka et al. (2008) found a positive relation between VE and circulating fibrinogen levels in teachers. Increased circulating fibrinogen levels have been linked to higher cardiovascular mortality and morbidity. These findings suggest that dysregulated hemostasis may be a biological pathway through which chronic stress may negatively impact cardiovascular health in teachers (Kudielka et al., 2008).

Vital exhaustion can be assessed subjectively with the Maastricht Vital Exhaustion Questionnaire (MQ; Appels and Mulder, 1989). However, researchers have been urged to aim for multimethod approaches to assess chronic stress, to account for the distinction between self-reported and physiological chronic stress (Cohen, 1997). Self-reports may be influenced by various individual factors such as mood and personality traits like neuroticism (Christensen et al., 2019). In addition, some items used in neuroticism and subjective stress-related measures may be similar, resulting in common method variance and reduced discriminant validity. Physiological measures of stress over time, on the other hand, are generally less influenced by an individual’s immediate mood or personality and are not affected by recalling biases and social desirability (Gow et al., 2010; Stirratt et al., 2015; Christensen et al., 2019; Prado-Gascó et al., 2019). Hair cortisol concentration (HCC) analysis has been a valuable tool for chronic physiological stress measurement and has been increasingly used in the last decade (Russell et al., 2012; Stalder and Kirschbaum, 2012). Cortisol is the most common glucocorticoid (GC) in humans and an essential element of the hypothalamus-pituitary–adrenal (HPA) axis stress response. By driving the release of GCs, the HPA axis is a central player in maintaining homeostasis and alerting the organism to environmental changes (Herman and Cullinan, 1997). HCC provides a unique opportunity to quantify long-term systematic cortisol exposure retrospectively over several months (Gow et al., 2010; Stalder and Kirschbaum, 2012; Ullmann et al., 2016). Circadian rhythms and acute changes regulated by the HPA axis are thought to affect HCC less than salivary cortisol (Stalder and Kirschbaum, 2012), and several studies showed significantly higher HCC in chronically stressed individuals (Dettenborn et al., 2010; Manenschijn et al., 2011; Stalder et al., 2017; Brianda et al., 2020).

To the best of our knowledge, no study has investigated the relationship between workaholism and neuroticism with HCC in teachers. However, some studies have linked HCC to work stress, with inconsistent results (Steinisch et al., 2014; Gidlow et al., 2016; Janssens et al., 2017; Herr et al., 2018). Regarding neuroticism and cortisol, previous studies have found an association between neuroticism and higher salivary basal cortisol or area under the curve on one or more days (e.g., Nater et al., 2010; Garcia-Banda et al., 2011; Laceulle et al., 2015). In contrast to salivary cortisol, research on the relationship between neuroticism and HCC is still scarce, but with promising results: Rietschel et al. (2016) reported a modest positive association between neuroticism and long-term HCC over the past three months. A study by Engert et al. (2021) showed neuroticism predicted HCC in 1 cm hair segments. Similarly, Erickson et al. (2021) found that neuroticism predicted higher HCC over 2 months, even after controlling for other personality traits. These results support using HCC to investigate associations between chronic physiological stress indicators and traits like neuroticism.

The aim of this longitudinal study is twofold. First, we intend to examine the relationship between workaholism and teachers’ chronic stress indicators, that is, self-reported vital exhaustion (VE) and hair cortisol concentrations (HCC; measured over three-cm hair segments) at two time points 1 year apart. Second, we aim to test the moderating role of neuroticism on the relationship between workaholism and VE and workaholism and HCC. Teachers have been found to have high levels of occupational stress and are at increased risk for burnout syndrome (Gluschkoff et al., 2016; Richards et al., 2018; García-Carmona et al., 2019). Examining long-term relationships between individual teacher characteristics and chronic stress-related indicators may provide a better understanding of contributing factors to high stress levels and psychosomatic complaints among teachers.

To tackle the study’s main objectives, we tested the following four hypotheses: (H1) Workaholism and neuroticism are associated with VE at t1 and t2 1 year later. Studies have found associations between workaholism and adverse subjective health outcomes, especially burnout (Ng et al., 2007; Andreassen, 2014; Clark et al., 2016). As VE may be considered a consequence of chronic stress and burnout (Bellingrath et al., 2008; Schoch et al., 2018), it seems reasonable to expect a positive association between workaholism and VE at t1 and t2. Neuroticism is considered a risk factor for burnout (Schaufeli and Enzmann, 2020). Thus, we expect to see an association between neuroticism and VE at t1 and, since it is a relatively stable personality trait (Borkenau and Ostendorf, 1993), likewise with VE at t2 1 year later. (H2) Neuroticism moderates the relationship between workaholism and VE at t1 and t2. This hypothesis is based on the assumption that a workaholic teacher with high neuroticism may be more vulnerable to psychological stress (Costa and McCrae, 1992). Such a teacher may also experience more self-doubt, anxiety, and pressure to perform than a workaholic teacher with low neuroticism (Clark et al., 2016; Aziz et al., 2018) and may consequently be at higher risk of experiencing VE. (H3) Workaholism and neuroticism are positively associated with HCC at t1 and t2. Neuroticism and workaholism appear to be risk factors for chronic stress (Clark et al., 2016; Erickson et al., 2021), which in turn has been associated with higher HCC (Russell et al., 2012; Stalder and Kirschbaum, 2012). (H4) Neuroticism moderates the relationship between workaholism and HCC at t1 and t2. Based on the literature above, we assume that workaholic teachers with high levels of neuroticism may have higher HCC than those with low neuroticism.

## Materials and methods

### Participants

School administrations were informed *via* flyers and emails, and the consent of the participating teachers was obtained. The inclusion criterion for participation in the study was an occupation as a primary or secondary teacher in Switzerland and a workload of a minimum of 16 lessons per week (equivalent to at least a 60 percent occupation). Fourty-two apparently healthy teachers (28 females, *M*_age_ = 39.66, SD = 11.99) completed the first online questionnaire on workaholism, neuroticism, and vital exhaustion. On average, the 42 teachers came from 39 different schools within the canton of Bern, Switzerland and had 13.35 years of teaching experience (SD = 11.07, range = 1–40). Participants had completed teacher training and were regular classroom teachers, not gifted or special education teachers. The grade levels they taught ranged from kindergarten and elementary school (1st to 6th grade; *n* = 27) to secondary school (7th, 8th, and 9th grade; *n* = 12) and to high school and vocational school (10th, 11th, and 12th grade; *n* = 3).

Three participants were either bald or had too short hair to take a hair sample, resulting in 39 teachers providing physiological data at the first measurement. When reaching the follow-up measurement 1 year later (t2), three more teachers withdrew their participation due to moving abroad (*n* = 1) and pregnancy (*n* = 2), resulting in 39 teachers with a follow-up self-report and 36 teachers with a follow-up self-report as well as follow-up physiological data.

### Procedure

All teachers were screened in a short interview to ensure that they met inclusion criteria. Participating teachers then signed informed consent. Subsequently, they completed online questionnaires on workaholism, neuroticism, and vital exhaustion and came to the University of Bern, Switzerland, where hair samples were taken at the beginning of 2020 before the COVID-19 Pandemic. One year after the first measurement (t2) during the COVID-19 Pandemic, participants completed another questionnaire on vital exhaustion and provided another hair sample. During data collection, teachers were teaching in their regular schools, not digitally.

The study was approved by the ethics committee of the canton of Bern and the Internal Review Board (IRB) of the University of Bern and was conducted in strict compliance with current data protection laws.

### Materials

#### Workaholism

To assess workaholism, we used subscale three (“Willingness to work until exhausted”) of the established inventory AVEM in its original German version (Schaarschmidt and Fischer, 2008). The English version was named the Measure of Coping Capacity Questionnaire (MECCA). Participants rated the six items with a value between 1 = not at all and 5 = a lot, depending on how much the items applied to them. Sample items were “If necessary, I work until exhaustion,” “I probably work more than I should,” and “I tend to work beyond my strength.” This resulted in a total sum score ranging from 6 to 30 points. The reliability was *α* =0.84.

#### Neuroticism

Neuroticism was assessed with two items of the short version of the Big Five personality trait scale (BFI-10; Rammstedt and John, 2007) and two additional items with the highest factor loadings of the NEO-FFI (Borkenau and Ostendorf, 1993). The structure of the combined items was tested using exploratory factor analysis. Both Bartlett’s test (*p* < 0.001) and the Kaiser-Meyer-Olkin measure of sampling adequacy (KMO = 0.675) indicate that the variables are suitable for factor analysis. Therefore, a Principal Component Analysis (PCA) with varimax rotation was performed. The one-factorial solution we obtained explained 56% of the variance. Participants indicated the extent to which each item described them, from 1 = not at all to 5 = a lot. Items were “I get depressed quickly, dejected” (NEO-FFI), “I worry a lot” (NEO-FFI), “I see myself as someone who is relaxed, handles stress well” (reversed item, BFI-10), and “I see myself as someone who gets nervous easily” (BFI-10). Mean values were calculated. The reliability of the four-item scale was α =0.73.

#### Vital exhaustion

VE was assessed at t1 and t2 (one-year follow-up) with the German translation (*cf.* Rau et al., 2010) of the Maastricht Vital Exhaustion Questionnaire (MQ; Appels and Mulder, 1989). The scale consists of 21 items assessing fatigue, difficulties falling asleep, general malaise, apathy, irritability, energy loss, depression, and waking up exhausted. Sample items were: “Do you sometimes feel as if your body is like a battery that is losing its power?,” “Do you have increasing difficulty in concentrating on a single subject for long?” “Do you often feel tired?.” Each of the 21 items could be rated on a three-point scale, from “statement is not true” (1) to “undecided” (2) or “true” (3). By summing the scores of each item, the total score could be calculated, with higher scores indicating increased vital exhaustion. The reliability was α =0.88 at t1 and α =0.88 at t2.

#### Hair cortisol concentration

Participants came to the University of Bern, where hair strands were cut from the posterior vertex as close to the scalp as possible. Hair cortisol concentrations were determined from the three-centimeter segment closest to the scalp. Given an average hair growth of 1 cm per month (Wennig, 2000), this segment represents the cumulative cortisol secretion over 3 months before sampling. The washing procedure and cortisol extraction followed the laboratory protocol described by Gao et al. (2013). All samples were analyzed by liquid chromatography coupled with tandem mass spectrometry (LC–MS/MS). This analysis’s lower limits of quantification (LOQ) were below 0.1 pg./mg for cortisol. The inter-and intra-assay coefficients of variance for cortisol were below 15% (Gao et al., 2013).

### Data analyses

Descriptive statistics and bivariate correlations were computed to investigate the study’s variables. The moderations were tested with ordinary least square regression models using SPSS 28 with the macro PROCESS version 4.0 (Hayes, 2018). The Shapiro–Wilk test was used for each variable to test whether the data were normally distributed. HCC at t1 and t2 were log-transformed due to non-normal distribution. Therefore, all correlations and regressions involving these two variables were calculated and interpreted using this transformation. The interaction terms were computed as the product of the mean-centered variables (except sex) involved. Significant interaction terms were followed up by simple slope tests at low (16th), middle (50th), and high (84th) percentiles of the moderator (Hayes, 2018). Participants’ sex, years of work experience, and the number of lessons taught per week were controlled for in the models. Only sex was associated with the predicted outcomes. Because of the small sample size, we preferred a parsimonious model and presented the results only with the control variable sex.

## Results

### Associations between self-reports and hair cortisol concentration

Descriptive characteristics and intercorrelations between workaholism, neuroticism, VE (t1 and t2), HCC (t1 and t2), and the control variables are shown in Table 1. In our sample, female teachers reported more VE at t2 (*r* = −0.36, *p* = 0.023) and had fewer years of work experience than their male colleagues (*r* = 0.39, *p* = 0.010). Both workaholism and neuroticism correlated significantly positively with VE at t1 as well as t2. Self-reports were unrelated to HCC at t1 and t2.

**Table 1:** Descriptive statistics and intercorrelations of the study variables.

Variable	*M*	SD	1.	2.	3.	4.	5.	6.	7.	8.
1. Sex^a^	−	−	−							
2. Work experience (years)	13.35	11.07	0.39**	−						
3. Lessons (per week)	23.07	3.97	−0.06	−0.23	−					
4. Workaholism	16.86	4.77	−0.01	−0.08	0.19	−				
5. Neuroticism	2.35	0.74	−0.10	−0.07	−0.04	0.55***	−			
6. Vital exhaustion t1	31.48	8.71	−0.18	−0.10	0.17	0.72***	0.73***	−		
7. Hair cortisol t1	7.83	7.62	0.17	0.08	0.24	0.20	0.09	0.10	−	
8. Vital exhaustion t2	32.23	7.95	−0.36*	−0.25	0.12	0.61***	0.68***	0.79***	0.01	−
9. Hair cortisol t2	5.77	4.23	0.15	−0.10	0.21	0.05	−0.07	−0.08	0.52***	−0.20

*N* = 42 (28 females), hair cortisol, and vital exhaustion T2: *N* = 39, hair cortisol T2: *N* = 36.

*p < 0.05, **p < 0.01, ***p < 0.001, two-tailed.

a Sex: 0 = female; 1 = male.

### Prediction of vital exhaustion by workaholism and neuroticism

Table 2 shows the results of the multiple regression analysis predicting VE at t1 and t2.

**Table 2:** Results of the multiple regression analyses predicting vital exhaustion t1 (*N* = 42) and t2 (*N* = 39).

Predictor	Vital exhaustion t1		Vital exhaustion t2	
	Model 1	Model 2	Model 3	Model 4
Sex^a^	−0.13	−0.26	−0.25*	−0.53**
Vital exhaustion t1	-	-	0.50**	0.31
Workaholism	0.47***	0.42***	0.09	0.16
Neuroticism	0.46***	0.45***	0.23	0.27^t^
Workaholism* Neuroticism	-	0.23**	-	0.22*

t1 = time 1; t2 = time 2. Standardized coefficients are reported. *R*^2^_Model 1_ = 0.70; *R*^2^_Model 2_ = 0.75; *R*^2^_Model 3_ = 0.70; *R*^2^_Model 4_ = 0.74.

*^t^p* < 0.10, *p < 0.05, **p < 0.01, ***p < 0.001, two-tailed.

a Sex: 0 = female; 1 = male.

*H1*: Model 1 shows that both workaholism (*β* = 0.47, *p* < 0.001) as well as neuroticism (*β* = 0.46, *p* < 0.001) were positively associated with VE at t1 when controlling for the participants’ sex (*β* = −0.13, *p* = 0.156). Both predictors together explain 70% of the variance [*F*(3, 39) = 29.10, *p* < 0.001, *R*^2^ = 0.70]. Model 3 shows the results of the multiple linear regression predicting VE at t2. Both workaholism t1 (*β* = 0.09, *p* = 0.559) and neuroticism t1 [*β* = 0.23, *p* = 0.112] were not significantly associated with VE at t2, when controlling for prior VE [*β* = 0.50, *p* = 0.006; *F*(4, 34) = 19.61, *p* < 0.001, *R*^2^ = 0.70]. There was a significant effect of sex, with female teachers reporting higher degrees of VE at t2 than male teachers (*β* = −0.25, *p* = 0.014).

**Figure 1:**
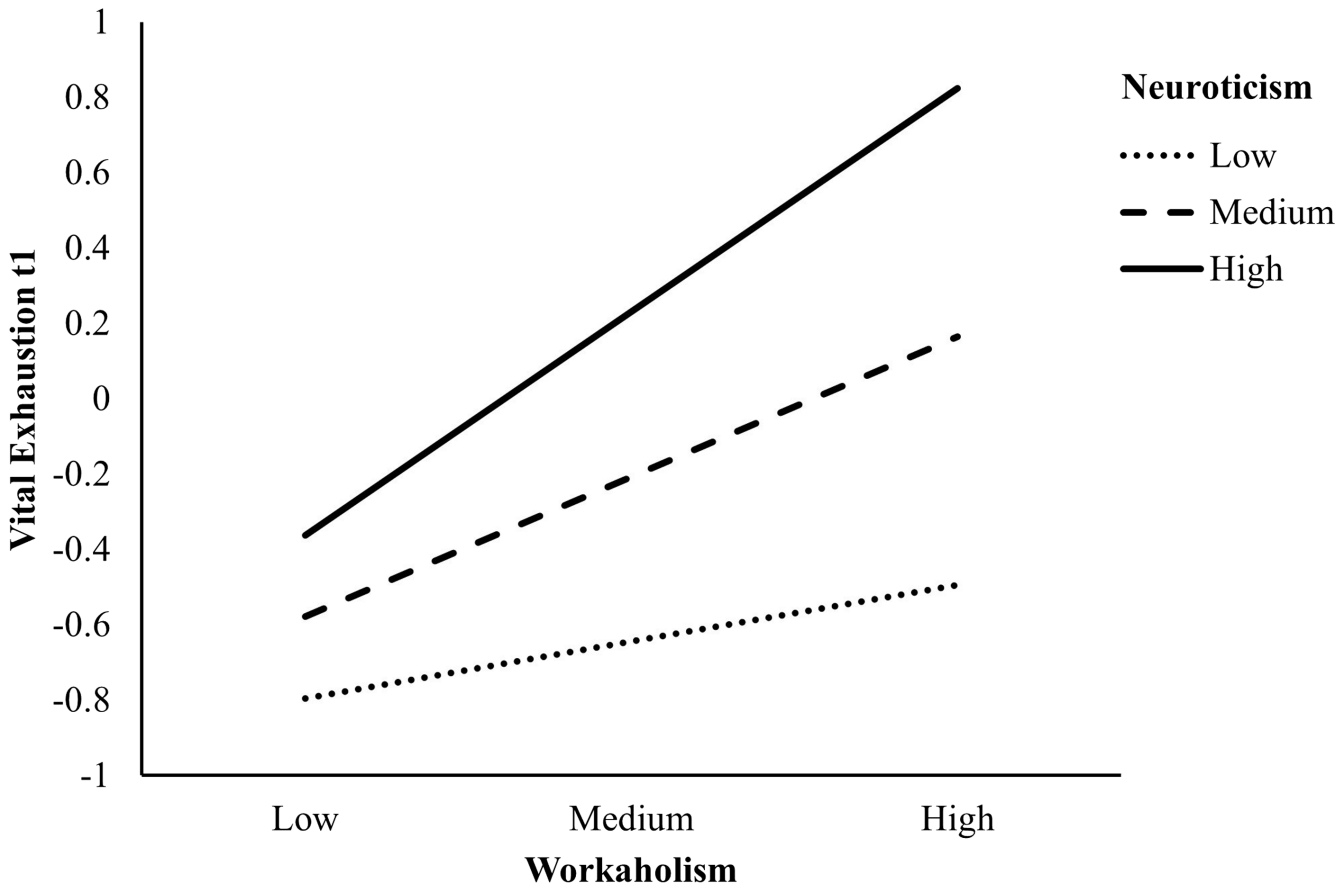
Interaction between workaholism x neuroticism on vital exhaustion at t1, controlling for sex (*N* = 39).

*H2*: Next, we analyzed the moderating role of neuroticism on the relationship between workaholism and VE at t1 and t2. The moderation model [model 2; *F*(4, 37) = 28.35, *p* < 0.001, *R*^2^ = 0.10] showed a significant interaction effect (*β* = 0.23, *p* = 0.006) when controlling for sex, which is shown in Figure 1. For teachers high in neuroticism, workaholism is positively associated with VE at t1 (simple slope: *β* = 0.62, SE = 0.11, *p* < 0.001), while for teachers low in neuroticism, there was no such association (simple slope: *β* = 0.16, SE = 0.15, *p* = 0.283).

**Figure 2:**
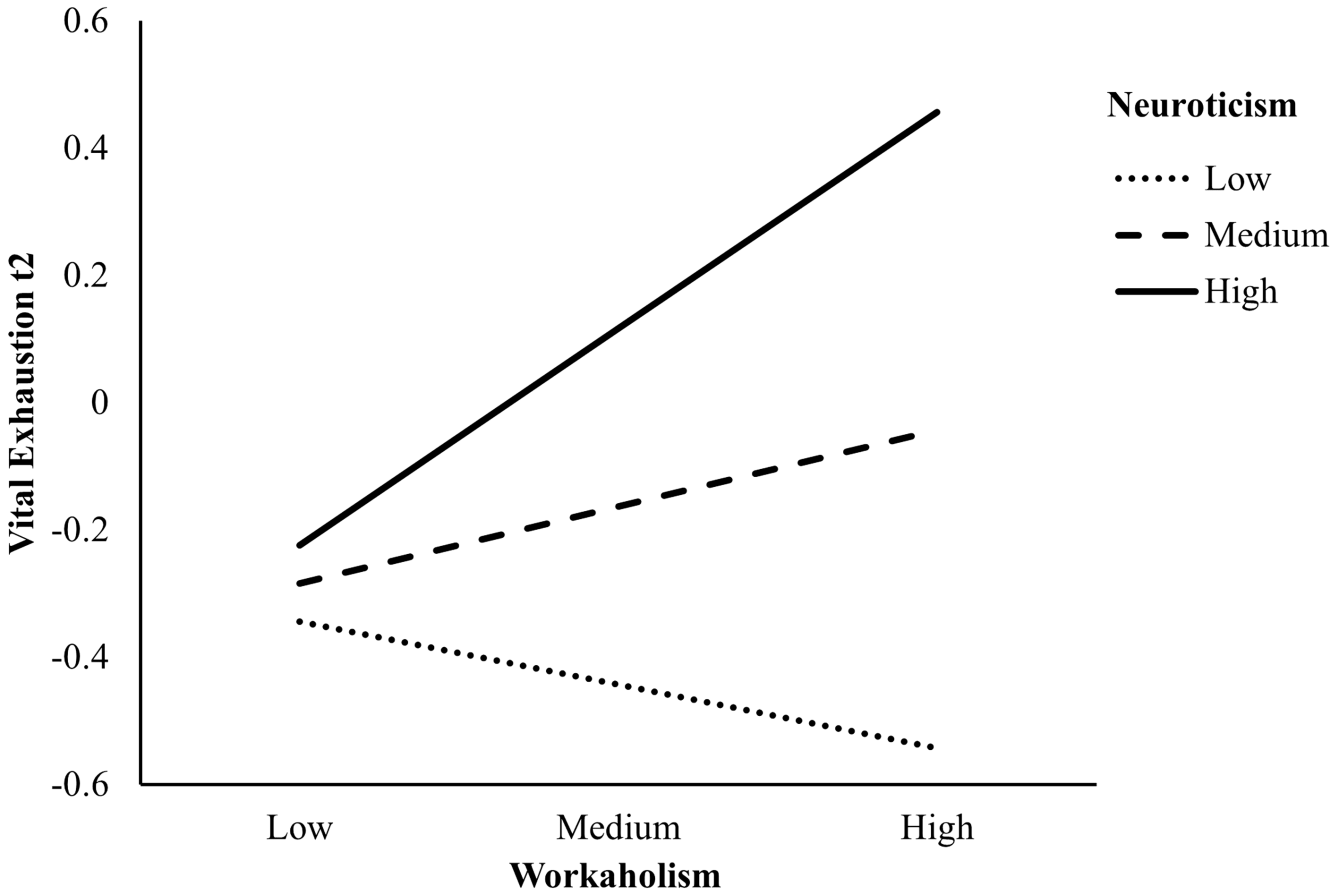
Interaction between workaholism x neuroticism on vital exhaustion at t2, controlling for vital exhaustion at t1 and sex (*N* = 39).

Model 4 examined the interaction between workaholism and neuroticism on VE at t2 [*F*(5, 33) = 18.84, *p* < 0.001, *R*^2^ = 0.74]. When controlling for VE t1 and sex, the interaction effect was significant as well (*β* = 0.22, *p* = 0.026). Figure 2 shows that for teachers high in neuroticism, workaholism is trend wise positively associated with VE t2 (simple slope: *β* = 0.35, SE = 0.18, *p* = 0.056), while for teachers low in neuroticism, there was no such association (simple slope: *β* = −0.10, SE = 0.16, *p* = 0.520). One year later, high neuroticism in combination with high scores in workaholism predicted high VE as well.

### Association of workaholism and neuroticism with hair cortisol concentration

*H3*: A multiple regression was conducted to predict HCC at t1 from workaholism and neuroticism (Table 3, model 1). The results show that neither workaholism (*β* = 0.22, *p* = 0.260) nor neuroticism (*β* = −0.00, *p* = 0.981) were significant predictors of HCC at t1, when controlling for gender (*β* = 0.19, *p* = 0.263; *F*(3, 35) = 0.95, *p* = 0.429, *R*^2^ = 0.08). The same multiple regression was calculated for HCC at t2 [model 3; *F*(4, 31) = 3.27, *p* < 0.05, *R*^2^ = 0.30]. Therefore, neither workaholism (*β* = 0.09, *p* = 0.661) nor neuroticism (*β* = −0.19, *p* = 0.316) were significant predictors of HCC 1 year later, when controlling for gender (*β* = 0.07, *p* = 0.677) and HCC at t1 (*β* = 0.52, *p* = 0.002).

*H4*: Models 2 and 4 explored whether there was a moderation of neuroticism on the association between workaholism and HCC at t1 and t2. However, no such effects were found.

**Table 3:** Results of the multiple regression analyses predicting hair cortisol (*N* = 39) and t2 (*N* = 36).

Predictor	Hair cortisol t1		Hair cortisol t2	
	Model 1	Model 2	Model 3	Model 4
Sex^a^	0.19	0.20	0.07	0.04
Hair cortisol t1	−	−	0.52*	0.52**
Workaholism	0.22	0.20	0.09	0.09
Neuroticism	−0.00	−0.00	−0.19	−0.17
Workaholism* Neuroticism	−	0.09	−	−0.21

t1 = time 1; t2 = time 2. *R*^2^_Model 1_ = 0.08; *R*^2^_Model 2_ = 0.08; *R*^2^_Model 3_ = 0.30; *R*^2^_Model 4_ = 0.34. Standardized coefficients are reported.

^t^*p* < 0.10, *p < 0.05, **p < 0.01, ***p < 0.001, two-tailed.

a Sex: 0 = female; 1 = male.

## Discussion

The present study aimed to investigate the interaction between the two potential risk factors of workaholism and neuroticism as longitudinal predictors of vital exhaustion (VE) and hair cortisol concentration (HCC). Additionally, we explored the moderating role of neuroticism on the relationship between workaholism, VE, and HCC.

The results partly confirmed our first hypothesis (H1), which stated that workaholism and neuroticism were positively associated with VE at t1. Our findings are consistent with previous research suggesting that workaholism and neuroticism may be risk factors for adverse subjective health outcomes (Preckel et al., 2005; Ng et al., 2007; Andreassen, 2014; Clark et al., 2016). The association between workaholism and VE supports the assumption that the exhaustive and compulsive work-related behaviors of workaholism come at the expense of teachers’ health and well-being (Preckel et al., 2005; Nie and Sun, 2016). However, contrary to our expectation, workaholism and neuroticism were both unrelated to VE one year later at t2 when controlling for t1. VE showed a very high longitudinal stability of *r* = 0.79 over the year between the two measurements at t1 and t2. Thus, not much variance remained that other variables could explain. Direct analysis at t2 without controlling for t1 showed similar effects as at t1. Furthermore, our results showed sex differences in VE at t2. One explanation could be that women generally report higher VE than men, which may be due to psychological and physiological sex differences in the stress response (Frestad and Prescott, 2017). In addition, studies suggest that women had a more significant decrease in mental health during the COVID-19 lockdown than men (Adams-Prassl et al., 2020), which may also have influenced the sex differences in our sample, as the follow-up data collection 1 year later occurred during the pandemic.

The second hypothesis stated that neuroticism moderates the relationship between workaholism and VE at t1 and t2, which has been confirmed. One possible explanation for this finding may be that teachers with high neuroticism scores are more likely to push themselves to or beyond the limits of their work performance, which eventually results in higher VE. Several previous studies support this assumption that neuroticism positively relates to workaholism as it may create an obsessive need or dependence on work, which, in turn, can result in adverse health outcomes (Lahey, 2009; Clark et al., 2016; McIlveen and Perera, 2016; Schaufeli, 2016, Gillet et al., 2018). In addition, neuroticism was found to be associated with low self-esteem (Amirazodi and Amirazodi, 2011), and some researchers suggest that one driving force behind workaholic employees may be the desire for self-worth validation (Aziz et al., 2018). Workaholics who experience low self-esteem, high levels of guilt, anxiety, and excessively high expectations of themselves are also likely to experience high levels of work stress (Clark et al., 2016; Aziz et al., 2018). High work stress combined with neuroticism may cause teachers to devote even more effort and time to work, perpetuating the vicious cycle.

Regarding hair cortisol concentrations (HCC), no association was found with workaholism or neuroticism, neither at t1 nor t2 (H3). One possible explanation for the associations of workaholism and neuroticism with vital exhaustion but not with HCC could be a simple negativity effect, that is, individuals high in neuroticism do not necessarily feel more exhausted or experience more workaholism, but they may report their feelings as stronger. Previous work suggests that neuroticism is associated with increased reporting of somatic symptoms but not with increased experience of these symptoms (McCrae and Costa, 1986; Larsen, 1992). In addition, the relationship between HCC and subjective work stress has yielded contradictory results in the existing literature (Qi et al., 2014, 2015; Steinisch et al., 2014; Gidlow et al., 2016; Janssens et al., 2017; Herr et al., 2018). Possible reasons may be the heterogeneity of the study populations, different hair sample lengths, and different stress questionnaires for different periods (Staufenbiel et al., 2013; Gidlow et al., 2016). Finally, van der Meij et al. (2018) compared HCC in a high and a normal workload sample and found an association with HCC only in the high workload sample. These findings suggest that a certain stress threshold must be reached to cause changes in HCC. The teachers in our sample are in the middle range between mild and moderate VE levels. Therefore, another reasonable explanation for our null findings may be that our teacher sample did not reach the threshold of chronic stress exposure that was high enough to associate with HCC.

The present study has several limitations. First, due to the COVID-19 pandemic, there were changes in the everyday lives of our participants, such as lockdowns and adaptation to digital teaching, which may have affected the stress-related measurements at one-year follow-up. The first assessments were conducted before the pandemic. Second, the assessment of neuroticism can be criticized since we did not use the full 12-item scale of the NEO-FFI (Borkenau and Ostendorf, 1993) which may not capture neuroticism in its broader concept. Third, workaholism and neuroticism were measured only once, which does not allow an analysis of the constructs’ bidirectional associations. Fourth, studies that have examined the overlap between vital exhaustion and depression have yielded inconsistent results regarding the extent to which they reflect the same underlying mechanisms (van Diest and Appels, 1991; Kopp et al., 1998; Kudielka et al., 2004; Vroege et al., 2012). Though our sample consists of generally healthy teachers, we did not control for depression, which may be considered a limitation. Finally, the sample of this study is relatively small, limiting the generalizability of the results. The findings should be replicated in a large cohort study to determine the effect of workaholism and neuroticism on adverse health outcomes in the general population. This would also help determine under what circumstances a relationship between workaholism, personality traits, and hair cortisol concentrations does or does not exist. That said, this study also includes some considerable strengths. This study is (to the best of the authors’ knowledge) the first to examine workaholism and neuroticism in relation to long-term hair cortisol concentrations in teachers. The longitudinal design further underscores the value of the study’s results.

If replicated in future research, our results have practical implications. In particular, the finding that workaholism was positively associated with vital exhaustion in teachers with high levels of neuroticism may highlight the importance of raising teachers’ awareness of potential risk factors contributing to vital exhaustion. In regards to neuroticism, a meta-analysis (Jorm, 1989) has shown that rational-emotive and similar therapies can successfully reduce neuroticism levels despite the dispositional nature of neuroticism. Moreover, providing in-service training courses for teachers may help familiarize them with the concepts of work behaviors, vital exhaustion, and the role of their personality in these processes. Although such training alone is unlikely to be sufficient to ultimately reduce the burden on teachers, it may help develop an understanding of potential adverse individual and organizational consequences of maladaptive or dysfunctional behaviors and coping styles. Identifying coping styles and work behaviors that match their personality style may help teachers to achieve and obtain a more sustainable work-life balance.

Taken together, workaholism and neuroticism appear to be associated with vital exhaustion in teachers. The association was manifested in increased self-reported vital exhaustion but not in increased HCC. Our findings emphasize the importance of considering individual characteristics when investigating vital exhaustion in teachers. Furthermore, this study adds to the literature on the divergence between self-reports and HCC. Further research is necessary to investigate the applicability of HCC as a biomarker of chronic stress in the context of work.

## Data availability statement

The raw data supporting the conclusions of this article will be made available by the authors, without undue reservation.

## Ethics statement

The studies involving human participants were reviewed and approved by Ethics Committee of the Canton Bern (no. 2019–00787) Internal Review Board (IRB) of the University of Bern. The participants provided their written informed consent to participate in this study.

## Author contributions

AW: conceptualization, methodology, formal analysis, and writing—review and editing. SS: conceptualization, investigation, and writing—original draft. GJ: formal analysis. MgH and WT: writing—review and editing. RLM: conceptualization, methodology, and writing—review and editing. All authors contributed to the article and approved the submitted version.

## Funding

This work was supported by the Swiss National Science Foundation SNF, under Grant number 100019_185484, and the University of Teacher Education Bern, Grant number 16 w 0008 02.

## Conflict of interest

The authors declare that the research was conducted in the absence of any commercial or financial relationships that could be construed as a potential conflict of interest.

## Publisher’s note

All claims expressed in this article are solely those of the authors and do not necessarily represent those of their affiliated organizations, or those of the publisher, the editors and the reviewers. Any product that may be evaluated in this article, or claim that may be made by its manufacturer, is not guaranteed or endorsed by the publisher.
